# Identifying early indicators of secondary peritonitis in critically ill patients with cirrhosis

**DOI:** 10.1038/s41598-021-00629-4

**Published:** 2021-10-26

**Authors:** Carole Ruault, Nathalie Zappella, Julien Labreuche, Pierrick Cronier, Baptiste Claude, Marc Garnier, Antoine Vieillard-Baron, Sofia Ortuno, Maxime Mallet, Olga Cosic, Laura Crosby, Olivier Lesieur, Nicolas Pichon, Arnaud Galbois, Cedric Bruel, Kenneth Ekpe, Bertrand Sauneuf, Damien Roux, Stephane Legriel

**Affiliations:** 1 Medical-Surgical Intensive Care Unit, Versailles Hospital, 177 rue de Versailles, 78150 Le Chesnay Cedex, France; 2 Anesthesiology and Critical Care Medicine Departement, DMU PARABOL, Bichat-Claude Bernard Hospital, HUPNVS, Assistance Publique-Hôpitaux de Paris (AP-HP), Paris, France; 3 Centre Hospitalier Régional et Universitaire de Lille, ULR 2694 - METRICS: Évaluation des Technologies de Santé et des Pratiques Médicales, 59000 Lille, France; 4 Intensive Care Unit, Sud-Francilien Hospital Center, 91100 Corbeil-Essonnes, France; 5 Department of Intensive Care, University Hospital François Mitterrand, 21000 Dijon, France; 6 Department of Anesthesiology and Critical Care Medicine, Sorbonne University, GRC 29, Assistance Publique-Hôpitaux de Paris (AP-HP), DMU DREAM, Tenon University Hospital, 75020 Paris, France; 7 Medical-Surgical Intensive Care Unit, Ambroise Paré University Hospital, APHP, 92100 Boulogne-Billancourt, France; 8 Medical Intensive Care Unit, Georges Pompidou European Hospital, Assistance Publique-Hôpitaux de Paris, 75015 Paris, France; 9 Groupe Hospitalier Universitaire APHP-Sorbonne Université, Site Pitié-Salpêtrière, Service de Pneumologie, Médecine Intensive et Réanimation (Département R3S), 75013 Paris, France; 10 Medical-Surgical Intensive Care Unit, Hôpital Nord Franche-Comté, 90400 Trevenans, France; 11 Intensive Care Unit, University Hospital of Pointe-à-Pitre, 97159 Pointe-à-Pitre, Guadeloupe France; 12 Intensive Care Unit, Groupement Hospitalier La Rochelle Ré Aunis, 17000 La Rochelle, France; 13 Medical-Surgical Intensive Care Unit, Limoges University Hospital, 87000 Limoges, France; 14 Ramsay-Générale de Santé, Hôpital Privé Claude Galien, Service de Réanimation Polyvalente, 91480 Quincy-sous-Sénart, France; 15 Medical and Surgical Intensive Care Unit, Groupe Hospitalier Paris Saint Joseph, 75014 Paris, France; 16 Medical Intensive Care Unit, Saint Louis Teaching Hospital, Assistance Publique Hôpitaux de Paris, 75010 Paris, France; 17 General Intensive Care Unit, Cotentin Public Hospital Center, 50100 Cherbourg-en-Cotentin, France; 18 Department of Intensive Care, Louis Mourier University Hospital, Assistance Publique-Hôpitaux de Paris, 92700 Colombes, France; 19 Université Paris-Saclay, UVSQ, CESP, Team DevPsy, 94807 Villejuif, Inserm France; 20 Present Address: Intensive Care Unit, Centre Hospitalier de Valence, 179 Boulevard Maréchal Juin, 26000 Valence, France

**Keywords:** Liver, Digestive signs and symptoms

## Abstract

Ascitic fluid infection (AFI) is a life-threatening complication of cirrhosis. We aimed to identify early indicators of secondary peritonitis (SP), which requires emergency surgery, and to describe the outcomes of SP and spontaneous bacterial/fungal peritonitis (SBFP). Adults with cirrhosis and AFI admitted to 16 university or university-affiliated ICUs in France between 2002 and 2017 were studied retrospectively. Cases were identified by searching the hospital databases for relevant ICD-10 codes and hospital charts for AFI. Logistic multivariate regression was performed to identify factors associated with SP. Secondary outcomes were short- and long-term mortality and survivors’ functional outcomes. Of 178 included patients (137 men and 41 women; mean age, 58 ± 11 years), 21 (11.8%) had SP, confirmed by surgery in 16 cases and by abdominal computed tomography in 5 cases. Time to diagnosis exceeded 24 h in 7/21 patients with SP. By multivariate analysis, factors independently associated with SP were ascitic leukocyte count > 10,000/mm^3^ (OR 3.70; 95%CI 1.38–9.85; *P* = 0.009) and absence of laboratory signs of decompensated cirrhosis (OR 4.53; 95%CI 1.30–15.68; *P* = 0.017). The 1-year mortality rates in patients with SBFP and SP were 81.0% and 77.5%, respectively (Log-rank test, *P* = 0.92). Patients with SP vs. SBFP had no differences in 1-year functional outcomes. This multicenter retrospective study identified two indicators of SP as opposed to SBFP in patients with cirrhosis. Using these indicators may help to provide early surgical treatment.

## Introduction

Cirrhosis is a common disease with an age-standardized incidence of 20.7/100,000 person-years and life-threatening complications responsible for 2 million deaths worldwide each year^1^. Ascites is the most common symptom of decompensation^2^ and can be complicated by primary spontaneous bacterial/fungal peritonitis (SBFP). SBFP results from pathogen translocation from the intestinal lumen to the peritoneal cavity in 10% to 30% of cases^3^. Rarely, patients with ascites develop secondary peritonitis (SP) due to perforation of an intra-abdominal organ, to an abdominal wall infection, or to paracentesis of the abdominal cavity.

The distinction between SBFP and SP is important, as SP nearly always requires emergent surgery. However, this distinction remains challenging to make. To date, only four studies have identified indicators of SBFP or SP^4–7^. A 1984 study in 38 patients suggested that SP was likely when the peritoneal fluid met at least two of the following three criteria: proteins > 1 g/dL, glucose < 50 mg/dL, and lactate dehydrogenase (LDH) > 225 mU/mL^5^. A decrease in ascitic neutrophil counts after 48 h has been reported to suggest SBFP. However, none of these studies focused specifically on patients managed in the intensive care unit (ICU)^4–7^.

Here, our primary objective was to identify factors associated with SP in patients with cirrhosis admitted to the ICU. Our secondary objectives were to describe mortality and functional outcomes. To achieve these objectives, we performed a multicenter retrospective observational study in 178 patients.

## Materials and methods

This study was approved by the French Health Authorities (*Comité d’Expertise pour les Recherches, les Etudes et les Evaluations dans le domaine de la Santé*, December 14, 2017, #TPS27472), which waived the requirement for written informed consent in accordance with French law on retrospective studies of anonymized data. Written information was delivered to all ICU survivors, who could then decline to participate in the study.

### Patients

Consecutive adults admitted between February 2002 and May 2017 to 16 university or university-affiliated ICUs in France with confirmed cirrhosis were identified by searching the hospital databases for International Statistical Classification of Diseases and Related Health Problems (ICD), 10th Revision (ICD-10), codes K70.3 (“Alcoholic cirrhosis of liver”), K71.7 (“Toxic liver disease with fibrosis and cirrhosis of liver”), and K74.X (“Fibrosis and cirrhosis of liver”). An additional search was performed on hospital charts using the term “cirrhosis”. Local investigators reviewed the medical records of the patients thus identified to select adults admitted to the ICU with ascitic fluid infection (AFI). All patients older than 18 years who were admitted to the ICU with SBFP or SP were included, except for those who underwent immediate surgery before ascitic fluid tests.

### Definitions

AFI was defined as the presence in the ascitic fluid of ≥ 250 polymorphonuclear leukocytes (PMNs)/mL, with or without a positive microbiological culture. SBFP was defined as AFI without any evidence of an intra-abdominal source of infection. SP was defined as a positive microbiological culture of ascitic fluid combined with imaging and/or surgery evidence of an intra-abdominal source of infection.

### Ascitic fluid infection (AFI) management

As recommended in current guidelines, SBFP was treated by draining the ascitic fluid and immediately administering empirical antimicrobial therapy. Selection of the antibiotics took into account the local ecology and whether the infection was acquired in the community or in the hospital. In most patients, a third-generation cephalosporin or fluoroquinolone was given, combined if appropriate with fluconazole or caspofungin. Additionally, patients received a human albumin infusion on day 1 and 3 of AIF^8^.

Patients with SP had emergency surgery, notably in the case of septic shock.

In all patients, the antimicrobial regimen was adapted as required by the results of the antimicrobial susceptibility tests on recovered microorganisms. In patients with shock, vasoactive drugs were given according to the hemodynamic monitoring data and response to a fluid challenge. Mechanical ventilation was used according to standardized criteria in patients with respiratory distress and/or coma, as well as in those requiring surgical management. Renal replacement therapy was given according to previously published criteria. All included patients required organ support^9^.

### Data collection

For each patient, a standardized form was used to collect demographic data, information on the history of cirrhosis (ascites, previous SBFP or SP, upper gastrointestinal bleeding, hepatic encephalopathy, Child–Pugh score, and other complications^10^); and the characteristics of the current AFI episode (circumstances of onset, clinical and laboratory features, and supportive treatments). We also collected the results of biochemical, cytological, and microbiological tests on ascitic fluid samples and blood culture bottles that were systemically bedside inoculated. The following data describing the ICU management were also collected: severity at ICU admission according to the Simplified Acute Physiology Score II (SAPS-II)^11^ The type(s) of organ failure(s) presented by the patients and motivating the admission to the ICU are reported here as coded in the patients' records. This characterization was not based on the use of a severity score. We also collected the need for mechanical ventilation, hemodynamic support, and/or renal replacement therapy during the ICU stay. Finally, the short- and long-term functional outcomes were assessed using the Glasgow Outcome Scale (GOS) score^12^ at hospital discharge then 3 and 12 months after ICU admission. We defined a favorable outcome as a GOS score of 4 or 5.

### Statistical analysis

Categorical variables were described as frequencies and percentages and continuous variables as mean ± (standard deviation, SD) if normally distributed and as median [interquartile range, IQR] otherwise. Normality of distributions was assessed graphically and by applying the Shapiro–Wilk test. Overall survival was estimated using the Kaplan–Meier method. Patients were divided into two groups according to the type of peritonitis, i.e., SBFP or SP. For comparisons of these two groups, Student’s *t* test was chosen for normally distributed continuous variables, the Mann–Whitney U test for nonnormally distributed continuous variables, and the chi-square test (or Fisher’s exact test when the expected sample size was < 5) for categorical variables.

To identify associations between patient characteristics and SP, we built a multivariable logistic regression model using Firth's penalized likelihood approach to account smaller number of SP^13^. Variables that were associated with SP with a P value < 0.05 by univariate analysis were entered into the model using forward stepwise selection. Before developing the multivariable model, we examined the log-linearity assumption for continuous variables (irrespective of results of previous univariate analyses) using restricted cubic spline functions. When the log-linearity assumption was not met, we used receiver operating characteristics (ROC) curve analysis to determine the variable that maximized the Youden index (Supplementary Fig. S1). This value was used to dichotomize the variable. To prevent collinearity, the following candidate variables were included in the multivariable analysis: abdominal pain at ICU admission, laboratory signs of decompensated cirrhosis (platelet count < 150 G/L and/or bilirubin > 50 µmol/L and/or prothrombin time < 40%), Runyon’s criteria, and an ascitic fluid nucleated cell count ≥ 10,000/mm^3^. The definition of laboratory signs of decompensated cirrhosis we used was based on the following pre-defined criteria: the lower limit of the pathological range for the platelet count, and the worst cut off values of the Child Pugh score for bilirubin and prothrombin time. The results of the multivariable analysis were expressed as odds ratios (ORs) of having SP, with their 95% confidence intervals (95%CIs). Performance of the model was assessed by testing calibration (using Hosmer–Lemeshow goodness-of-fit test) and discrimination (by computing the C-statistic). To avoid loss of patients for the multivariable analysis due to missing data for candidate variables, the multivariable analysis was performed after multiple imputation of missing values using a switching regression approach (chained equations with m = 10). Imputation was performed under the missing-at-random assumption, using all variables listed in Table 1, with a predictive mean matching method for continuous variables and a multinomial or binary logistic regression model for categorical variables. Estimates obtained in the different imputed data sets were combined using Rubin’s rules.

**Table 1:** Main characteristics of 178 patients with cirrhosis and spontaneous bacterial/fungal peritonitis (SBFP) or secondary peritonitis (SP).

Patient characteristics	N (%) or median [interquartile range] or Mean (± standard deviation)				*P* value
	SBFP		SP		
	n missing^†^	n = 157 (88.2%)	n missing^†^	n = 21 (11.8%)	
**Demographic characteristics**					
Age (years)		57.6 (± 10.8)		59.3 (± 10.1)	0.49
Males		124 (79.0)		13 (61.9)	0.081
Previous history of cirrhosis		128 (81.5)		16 (76.2)	0.56
Cause of cirrhosis					–
Alcohol-related*		126		17	
HCV*		24		2	
HBV*		9		0	
NASH*		7		1	
Other*		17		1	
Previous cirrhosis decompensation		105 (66.9)		11 (52.4)	0.19
Ascites		96 (61.2)		10 (47.6)	0.24
SBFP		18 (11.5)		4 (19.1)	0.32
Upper gastrointestinal bleeding		34 (21.7)		5 (23.8)	0.78
Hepatic encephalopathy		24 (15.3)		4 (19.1)	0.75
Child–Pugh score	13	11 [9–12]	2	10 [8–12]	0.25
SAPS II score on day 1	1	61.8 (± 21.5)	1	57.2 (± 21.3)	0.37
**Clinical findings and pre-hospital presentation**					
Glasgow Coma Scale score	5	14 (7–15)	3	14 (10–15)	0.69
Mean arterial pressure (mmHg)	4	72.2 (± 19.6)		79.5 (± 17.0)	0.11
Heart rate (beats/min)	6	99.1 (± 23.4)		100.0 (± 22.2)	0.86
Respiratory rate (breaths/min)	29	23.9 (± 6.7)	6	23.9 (± 6.5)	1.00
Temperature ≤ 36 or ≥ 38.5 °C	8	60 (40.3)	1	9 (45.0)	0.69
Abdominal pain		55 (35.0)		13 (61.9)	0.017
Hepatic encephalopathy			1		
None		89 (56.7)		14 (70.0)	0.22
Grade 1		23 (14.7)		1 (5.0)	
Grade 2		18 (11.5)		4 (20.0)	
Grade 3		12 (7.6)		1 (5.0)	
Grade 4		15 (9.6)		0	
Upper gastrointestinal bleeding		29 (18.5)		1 (4.8)	0.21
Prophylactic antibiotic therapy		30 (19.1)		2 (9.5)	0.38
**Laboratory findings**					
Bilirubin (µmol/L)	13	107 [48–180]	3	48 [23–98]	0.004
Albumin (µmol/L)	68	23.2 (7.6)	9	23.1 (4.1)	0.97
Creatinine (µmol/L)	8	182 [104–268]	3	178 [101–221]	0.60
Lactate (mmol/L)	37	4.9 [2.6–8.4]	6	3.3 [2.5–9.7]	0.53
Blood neutrophil count (/mm^3^)	39	10.7 [6.3–18.9]	7	12.6 [6.2–21.4]	0.68
Platelet count (G/L)	5	92 [58–142]	3	152 [42–336]	0.023
Prothrombin time (%)	14	34 (17)	3	42 (18)	0.048
Blood signs of decompensated cirrhosis^a^	7	68 (45.3)	3	3 (16.7)	0.020
**Ascitic fluid analysis**					
Protein (g/L)	29	11 [7–18]	3	20 [15–30]	< 0.001
≥ 14.8^b^		43 (33.6)		15 (83.3)	< 0.001
Glucose (mmol/L)	102	6.1 [3.4–7.4]	12	7.2 [0.1–8.0]	0.70
≤ 7.2^b^		41 (74.6)		5 (55.6)	0.25
LDH (IU/L)	123	210 (136–401)	11	632 [192–857]	0.042
≤ 500^b^		28 (82.4)		3 (30.0)	0.003
PMN count (/mm^3^)	2	2083 [611–5640]		2720 [952–10000]	0.10
≥ 7560^b^		29 (18.7)		9 (42.9)	0.020
Polymicrobial culture		14 (8.9)		5 (23.8)	0.054
Leukocyte count (/mm^3^)	9	2700 [854–6100]	3	4200 [2200–10500]	0.055
≥ 10,000^b^		27 (18.2)		8 (44.4)	0.026
Runyon’s criteria^c^	87	18 (25.7)	9	7 (58.3)	0.039

*SBFP* spontaneous bacterial/fungal peritonitis, *SP* secondary peritonitis, *SAPSII* Simplified Acute Physiology Score version II, *LDH* lactate deshydrogenase, *PMN* polymorphonuclear leukocytes.

^†^Number of missing observations. If ≥ 1.

*Some patients had more than one cause of cirrhosis.

^a^Defined as platelet count < 150 G/L and/or bilirubin > 50 µmol/L and/or prothrombin time < 40%.

^b^Dichotomization by maximizing the Youden index of the ROC curve.

^c^At least 2 ascitic fluid criteria among the following: protein > 10 g/L glucose < 2.7 mmol/dL and LDH > upper limit of normal in serum.

Finally, prognostic variables were compared between the SP and SBFP groups using the chi-square test for binary variables and the Mann–Whitney U test for 28-day mechanical ventilation-free days. For ICU and hospital stay lengths, we applied Gray’s test with mortality as a competing risk and application of the log-rank test for overall survival. Statistical testing was done at the two-tailed α level of 0.05. The statistical analysis was performed using the SAS software package, release 9.4 (SAS Institute, Cary, NC).

### Consent to participate (Ethics)

This study was approved by the French Health Authorities (*Comité d’Expertise pour les Recherches, les Etudes et les Evaluations dans le domaine de la Santé*, December 14, 2017, #TPS27472), which waived the requirement for written informed consent in accordance with French law on retrospective studies of anonymized data. All procedures involving the patients complied with the ethical standards of the institutional and national research committees and with the 1964 Declaration of Helsinki and its later amendments.

### Consent to publish

All authors consent to the publication of the manuscript.

## Results

Of the 6513 critically ill patients with cirrhosis managed in the 16 university and university-affiliated hospitals during the 16-year study period, 2373 had ascites. Among them, the 178 patients with SBFP or SP were included in the study.

### Baseline characteristics and ICU management

Table 1 reports the main patient characteristics. There were 137 men and 41 women with a mean age of 58 ± 11 years. A previous history of cirrhosis was noted in 144 (80.1%) patients, of whom 116/144 (80.6%) had experienced at least one decompensation episode: ascites in 106, SBFP in 22, upper gastrointestinal bleeding in 39, and hepatic encephalopathy in 28.

All but 28 patients demonstrated on-scene vital organ failure requiring ICU admission: the failure was hemodynamic in 91 patients, neurological in 28, respiratory in 22, renal in 5, and involved multiple organs in 4. The pre-hospital median Glasgow Coma Scale score was 14 [8–15], and 74 (41.8%) of patients showed signs of hepatic encephalopathy of any grade. Mechanical ventilation was required in 125 (70.2%) patients, catecholamines in 131 (73.5%), and renal replacement therapy in 51 (28.7%). Upper gastrointestinal bleeding was present in 30 (16.9%) patients, of whom 24/30 (80.0%) had a coagulopathy and received a blood product transfusion.

### Diagnosis of ascites fluid infection (AFI)

Paracentesis was performed 0 (0–1) day after ICU admission. At the time, mean body temperature was 36.0 ± 1.4 °C and abdominal pain was noted in 68 (38.2%) patients. Median PMN count was 11.2 [6.3–19.1]/mm^3^, C-reactive protein was 84 [40–139] mg/L, and procalcitonin was 4.9 [1.8–11.5] ng/mL. Antimicrobials had been administered before the paracentesis to 32 (18.0%) patients due to septic shock or concomitant upper gastrointestinal bleeding.

Ascitic fluid characteristics were as follows: protein 12 [7–19] g/L, glucose 6.2 [2.9–7.6] mmol/L, LDH 219 [139–577] IU/L, PMN count 2183 [645–6087]/mm^3^, and total leukocyte count 2785 [960–7000/mm^3^]. Table 2 shows the results of direct Gram staining and microbiological cultures of ascitic fluid samples. In the 99 (55.6%) patients with microbiological documentation, both the Gram stain and the cultures were positive. Of these 99 patients, 80/99 (80.8%) had a single micro-organism recovered and 19/99 (19.2%) more than one micro-organism. All but two patients received antibiotic therapy and 24 (13.5%) underwent emergent surgery, including 16 with SP and 8 with SBFP.

**Table 2:** Gram stain and microbiological culture findings in ascitic fluid samples from 178 patients with cirrhosis and spontaneous bacterial/fungal peritonitis (SBFP) or secondary peritonitis (SP).

Gram stain and microbiological culture findings	SBFP n = 157 (88.2%)	SP n = 21 (11.8%)
Negative direct Gram stain and culture	71 (45.2%)	8 (38.1%)
Polymicrobial	14 (8.9%)	5 (23.8%)
**Gram-negative bacilli**	46 (29.3%)	3 (14.3%)
Escherichia coli	34	3
Enterobacter cloacae	2	0
Klebsiella sp.	4	0
Pseudomonas aeruginosa	3	0
Other Gram-negative bacilli	3	0
**Gram-positive cocci**	21 (13.4%)	4 (19.0%)
Enterococcus faecalis	3	1
Enterococcus faecium	2	1
Other Enterococci	1	0
Streptococcus species	10	1
Staphylococcus aureus	4	1
Other Staphylococci	1	0
**Gram-positive bacilli**	2 (1.3%)	0
Clostridium perfringens	1	0
Listeria monocytogenes	1	0
**Fungi**	2 (1.3%)	1 (4.7%)
Candida glabrata	2	0
Candida albicans	0	1
Undetermined	1 (0.6%)	0

*SBFP* spontaneous bacterial/fungal peritonitis, *SP* secondary peritonitis.

### Diagnosis of spontaneous bacterial/fungal peritonitis (SBFP) and secondary peritonitis (SP)

Of the 178 patients, 157 (88.2%) received a final diagnosis of SBFP and 21 (11.8%) a diagnosis of SP. Of the 24 patients who underwent surgery, 8 had SBFP and no evidence of intra-abdominal infection and 16 had SP (duodenal ulcer perforation, n = 4; cholecystitis, n = 3; appendicitis, n = 2; small bowel or colon perforation, n = 2; salpingitis, n = 1; stomal suture insufficiency, n = 1; omphalocele complication, n = 1; and undetermined diagnosis, n = 2). In the remaining 5 patients with SP, the diagnosis was provided by abdominal CT (traumatic digestive tract puncture, n = 2; pancolitis, n = 1; chronic pancreatitis fistula, n = 1; and pneumoperitoneum of unknown etiology n = 1). Of the 21 patients with SP, 7 had the diagnosis of SP established after more than 24 h in the ICU.

### Factors associated with secondary peritonitis (SP)

By multivariate analysis, after multiple imputation for missing data, factors independently associated with SP were ascitic leukocyte count > 10,000/mm^3^ (OR 3.70; 95%CI 1.38–9.85; *P* = 0.009) and absence of laboratory signs of decompensated cirrhosis (OR 4.53; 95%CI 1.30–15.68; *P* = 0.017).

Table 3 reports the diagnostic performances (sensibility and specificity) of factors identified as independently associated with SP.

**Table 3:** Sensibility and specificity of independent factors of secondary peritonitis.

	Sensitivity	Specificity
**Individual prognostic values**		
Ascitic leukocyte count > 10,000 m^3^	9/21 (42.9)	129/157 (82.2)
Absence of laboratory sign of decompensated cirrhosis^a^	18/21 (85.7)	74/157 (47.1)
**Combined prognostic values**		
At least one criteria	9/21 (42.9)	145/157 (92.4)
Both criteria	18/21 (85.7)	59/157 (37.8)

Values are no./total no. (%) unless otherwise as indicated (calculated after handling missing values by multiple imputation).

^a^Defined as platelet count < 150 G/L and/or bilirubin > 50 µmol/L and/or prothrombin time < 40%.

### Short-term and long-term outcomes

**Figure 1:**
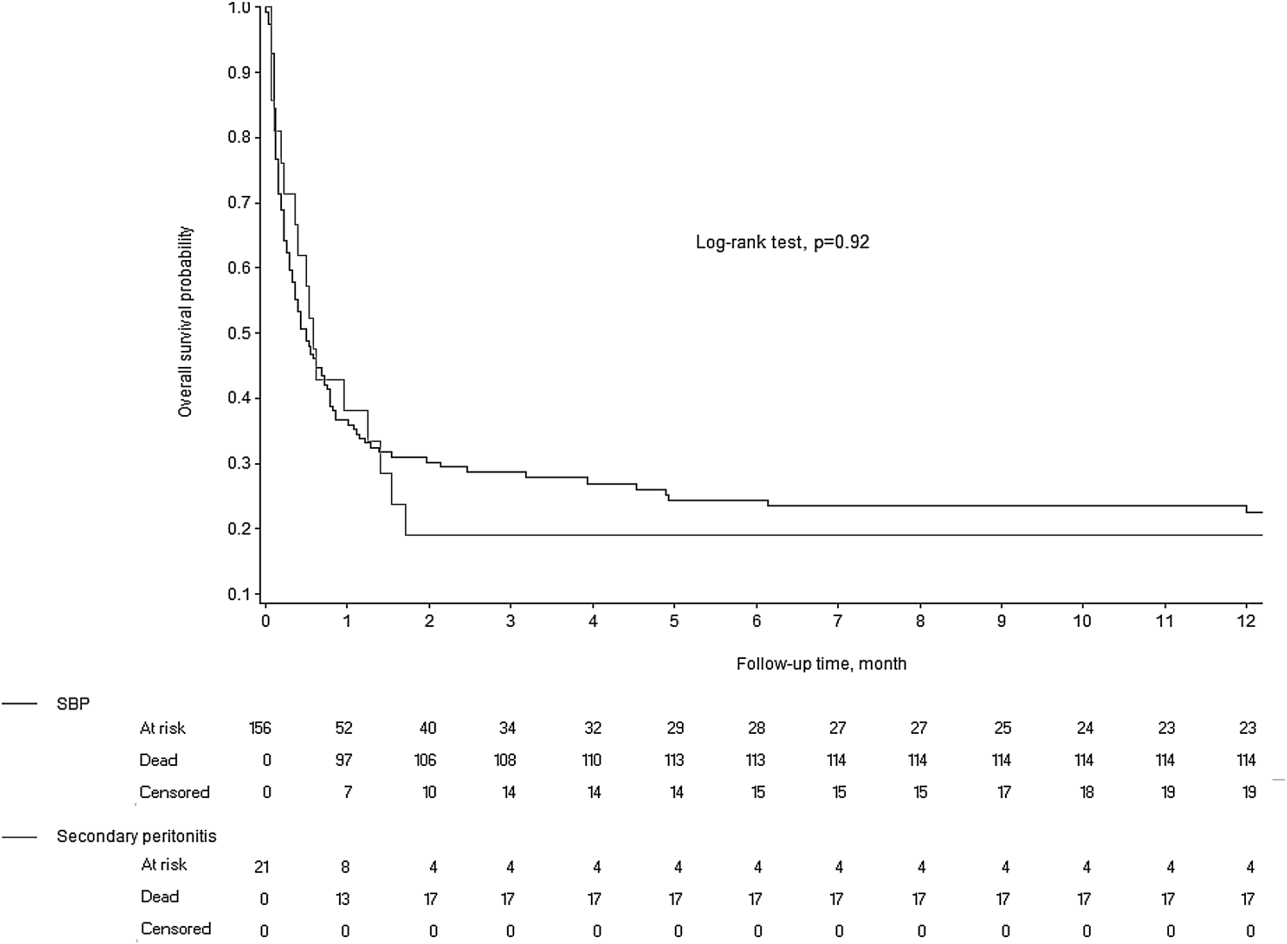
Kaplan–Meier estimates of overall survival in patients with spontaneous bacterial/fungal peritonitis (SBFP) and secondary peritonitis (SP).

Figure 1 shows the Kaplan–Meier survival analyses for 1-year mortality in the SP and SBFP groups. The 1-year mortality rate was 81.0% in the SP group and 77.5% in the SBFP group (Log-rank test, *P* = 0.92). In addition, all 5 patients with SP who were not treated surgically died during their hospital stay.

Table 4 reports the outcomes in the SP and SBFP groups. No differences were observed between groups regarding organ failure support and ICU and hospital lengths of stay. Overall, the functional outcome was poor in survivors at all follow-up time points, with no difference between the SP and SBFP groups.

**Table 4:** Outcomes of 178 critically ill patients with cirrhosis and spontaneous bacterial/fungal peritonitis (SBFP) or secondary peritonitis.

Outcomes	N (%) or Median [interquartile range]		*P* value
	SBFP n = 157 (88.2%)	SP n = 21 (11.8%)	
28-day mechanical ventilation-free days	2 [0–6]	2 [0–14]	0.27
Renal replacement therapy during the hospitalization	45/157 (28.7)	6/21 (28.6)	0.99
Catecholamines during the hospitalization	114/157 (72.6)	17/21 (81.0)	0.42
Bacteremia during the hospitalization	56/157 (35.7)	4/21 (19.1)	0.13
ICU length of stay (days)	6 [4–12]	12 [4–17]	0.96
Hospital length of stay (days)	12 [5–24]	18 [7–43]	0.18
**Good outcome (GOS 4 or 5)**			
At ICU discharge	35/156 (22.4)	7/21 (33.3)	0.28
At hospital discharge	35/150 (23.3)	4/21 (19.1)	0.79
At 3 months	29/144 (20.1)	4/21 (19.1)	1.00
At 1 year	24/139 (17.3)	3/20 (15.0)	1.00

*SBFP* spontaneous bacterial/fungal peritonitis, *SP* secondary peritonitis, *ICU* Intensive Care Unit, *GOS* Glasgow Outcome Scale.

## Discussion

This retrospective study included 178 critically ill patients with cirrhosis-related ascites and either SBFP or SP. Factors independently associated with SP were ascitic leukocyte count > 10,000/mm^3^ and absence of laboratory signs of decompensated cirrhosis. Survival and short- and long-term functional outcomes were poor but similar between the groups with SBFP and SP.

The patients’ characteristics were consistent with previous studies of patients with cirrhosis admitted to the ICU^9,14–17^. However, few studies have investigated critically ill patients with SBFP or SP^4–7^. Our population shares similarities with that in another retrospective study, with a mean age of approximately 60 years, a marked male predominance, and a majority of Child–Pugh C patients^6^. As previously reported, the main causes of cirrhosis were alcohol abuse, hepatitis C and B, and non-alcoholic steatohepatitis^1^. We therefore believe that our sample is representative of patients with cirrhosis-related AFI requiring ICU management.

In our study, the prevalence of AFI in the overall population of patients with cirrhosis admitted to the participating ICUs was 36.4%. Among patients with SBFP or SP, 44% had negative ascitic fluid Gram stains and cultures, in keeping with the range of 40% to 96.5% in previous studies^18^. In patients with positive ascitic fluid cultures, polymicrobial cultures predominated in patients with SP but were far less common in the group with SBFP. These findings are consistent with the underlying pathophysiology, since SBFP is chiefly caused by pathogen translocation from the bowel^19^, whereas SP is due to infection of the bowel and/or bile ducts. Surprisingly, the magnitude of this result is relative, however, since we identified only 24% of cases with a polymicrobial culture in patients with SP. This is in contrast to the classical understanding and observations. Anaerobes and fungi should always prompt for investigation of SP and are expected in patients with perforation. Moreover, this difference was not statistically significant, perhaps due to limited statistical power. Interestingly, *Escherichia coli* was the most frequently isolated pathogen, suggesting a higher propensity of this organism for translocation^2^. Spontaneous fungal infection was encountered in 1.3% of our patients^20^. Although the prevalence of fungal infection is extremely low in our series, the concept of spontaneous fungal infection is somewhat controversial. Importantly, none of the patients with fungal peritonitis had a plastic peritoneal catheter in place. A polymicrobial positive ascitic fluid culture was not significantly associated with SP in our population. Furthermore, SP requires emergent surgery, notably in patients with septic shock, which does not leave enough time to wait for culture results. In our population, emergent surgery was associated with a final diagnosis of SP in 8/24 cases. Finally, patients with SP experienced more significantly abdominal pain. The main hypothesis we can formulate is that abdominal pain could be related to the cause of the ascites infection itself, and not just to the abdominal distension related to the ascites volume. Thus, the higher rate of abdominal pain in patients with SP could be explained by the presence of duodenal ulcer perforation, small bowel or colon perforation and their related pneumoperitoneum, cholecystitis, appendicitis, , or even salpingitis. Finally, it is not possible for us to compare the volume of ascites in the two groups since some patients may have had only one exploratory puncture with surgery (in patients with SP) and the volume of ascites evacuated was then not measured.

We identified two factors independently associated with SP, namely, an ascitic fluid leukocyte count > 10,000/mm^3^ and absence of laboratory signs of decompensated cirrhosis. The elevated leukocyte count is consistent with the pathophysiology of SP. Bowel or bile-duct perforation releases a far larger inoculum of microorganisms into the peritoneal cavity, compared with bacterial translocation from the bowel. This large inoculum causes a stronger inflammatory reaction than that seen in SBFP^21^. Laboratory signs of cirrhosis decompensation were defined for our study as a platelet count < 150 G/L, bilirubin level > 50 µmol/L, and prothrombin time < 40%. The sudden onset of SP and its pathophysiological mechanisms described above may explain the discriminating nature of these laboratory abnormalities. Also, SP may occur independently of the stage of cirrhosis, whereas SBFP is a typical complication of ascitic decompensation with a drop in protein levels.

Interestingly, Runyon's criteria for SP (protein > 1 g/dL, glucose < 50 mg/dL, and LDH > 225 U/L) were rarely met in our population. However, the data needed to assess them were available in only 46% of our patients. Nevertheless, our statistical analysis used imputation techniques to handle missing data. Runyon’s criteria were developed several decades ago (in 1984), based on the analysis of only 38 patients, of whom only 6 had SP^5^. Runyon suggested that the criteria might be related to the large bacterial inoculum observed in SP, with marked glucose consumption by bacteria and an LDH increase reflecting the rapid metabolism of glucose^5^. The high protein level may be ascribable to tissue damage and inflammatory cell infiltration at the site of the bowel or bile-duct lesion in SP. The accuracy of Runyon’s criteria has been evaluated in several studies^4–7^. The largest was a retrospective study in 106 patients that demonstrated 66.6% sensitivity and 89.7% specificity. In our study, meeting Runyon’s criteria was not independently associated with SP.

Mortality was high in our population. Mortality in patients with cirrhosis admitted to the ICU is currently about 30%–50%^22^ but can reach 70% in patients with septic shock^9,14,17,23^. In patients admitted to standard medical wards, SBFP does not seem to increase mortality compared to patients with cirrhosis-related ascites admitted for other reasons^9^. However, our results are concordant with a previous retrospective study in which the ICU and hospital mortality rates of patients with cirrhosis and septic shock were considerably higher compared to patients with septic shock but no cirrhosis (70.1% and 74.5% vs. 48.3% and 51.7%, respectively). In addition, cirrhosis was independently associated with death in the ICU during septic shock^9^. We found no significant difference between the groups with SBFP and SP in terms of mortality or of favorable GOS scores in survivors at ICU and hospital discharge and after 3 months and 1 year. The time from ICU admission to surgery in SP has not yet been reported as associated with the prognosis. Nonetheless, early identification of SP leading to prompter surgery may improve patient outcomes^24,25^. Further studies are warranted to evaluate this possibility.

Our study has several limitations. First, the general applicability of our results to the overall population of critically ill patients with AFI remains to be confirmed. The patients in this study were admitted to 16 ICUs over a 16-year period, and their management may have varied across centers and over time. However, due to the low incidence of critically illness in patients with cirrhosis and AFI, the long inclusion period was necessary to obtain a sample large enough to provide the statistical power needed for identifying independent indicators of SP. Prospectively including the same number of patients would require a very large number of centers and a relatively long inclusion period that would also carry the possibility of changes in practices over time. We therefore chose a retrospective design. We regret not being able to provide these additional details which would have allowed us to better characterize our population. By example, two cases of AFI are attributed to the SP group because surgical findings indicated evidence of peritonitis although the precise cause of digestive perforation could not be characterized. As these findings were reported as this in the medical records and surgical reports and it seemed inappropriate to disregard them in the retrospective collection of data for this study. Unfortunately, the retrospective nature of our work does not allow the calculation of the MELD score or EF CLIF. However, we were able to report a Child Pugh score in 165 (93%) patients, which allows us to balance this lack of information. This also resulted in a substantial number of missing data, a fact that was taken into account by performing multiple imputations. Finally, we were unable to assess whether antimicrobial treatment resulted in a decrease in the ascitic fluid PMN count 48 h after the first paracentesis, as less than half our patients had a second paracentesis. In any case, SP requires emergent surgery and the diagnosis must therefore be suspected very early.

## Conclusion

In our population of critically ill patients with cirrhosis and AFI, SP was associated with an ascitic fluid leukocyte count > 10,000/mm^3^ and with absence of laboratory signs of cirrhosis decompensation. These new indicators may help to reduce the time to surgery in patients with SP. Further studies are needed to confirm these results and to determine whether using these two factors to suspect SP improves the currently poor prognosis.

## Supplementary Information

Supplementary Information.

## Data Availability

The data that support the findings of this study are available from the corresponding author, [SL], upon reasonable request.

## Abbreviations

AFI: Ascitic fluid infection
GOS: Glasgow Outcome Scale
ICD: International Statistical Classification of Diseases and Related Health Problems
ICU: Intensive care unit
IQR: Interquartile range
ORs: Odds ratios
PMNs: Polymorphonuclear leukocytes
ROC: Receiver operating characteristics
SAPS-II: Simplified Acute Physiology Score II
SBFP: Spontaneous bacterial/fungal peritonitis
SP: Secondary peritonitis
