# Supplementary material for: Identifying early indicators of secondary peritonitis in critically ill patients with cirrhosis
Source: Sci Rep. 2021 Oct 26;11:21076. doi: 10.1038/s41598-021-00629-4 (PMC8548403; doi:10.1038/s41598-021-00629-4)
Supplement: Supplementary file 1 — Supplementary Information. [file 41598_2021_629_MOESM1_ESM.pdf]

Supplementary Figure S1.

Panel A. Protein ascitic fluid count

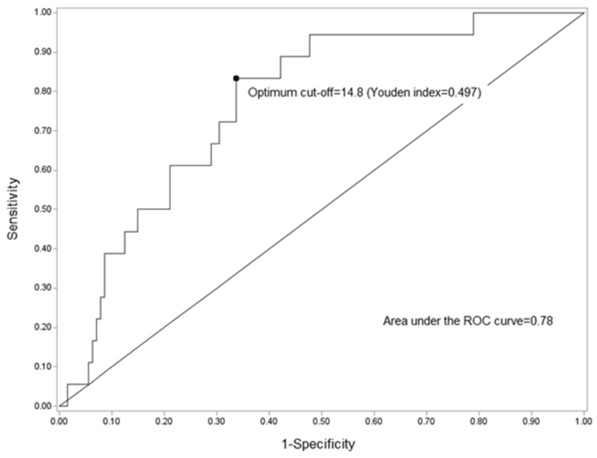

Panel C. Lactate Deshydrogenase ascitic fluid count

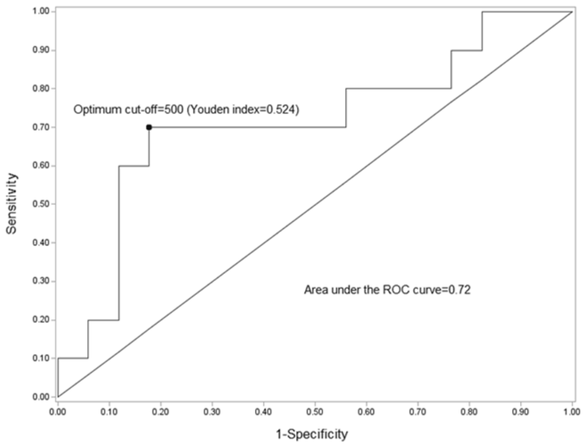

Panel E. Leukocyte ascitic fluid count

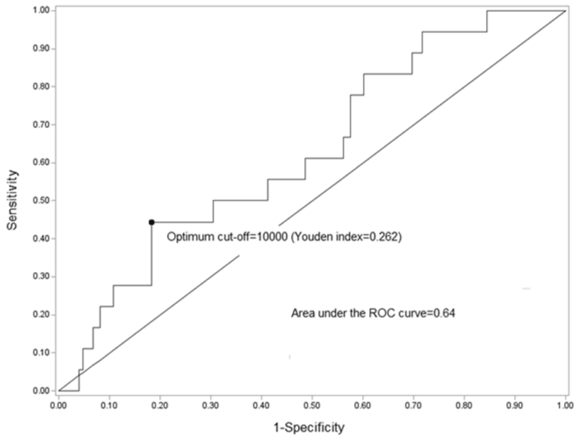

Panel B. Glucose ascitic fluid count

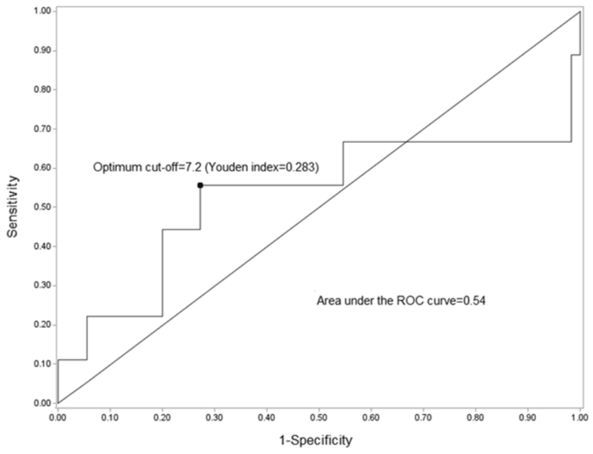

Panel D. polymorphonuclear leukocytes ascitic fluid count

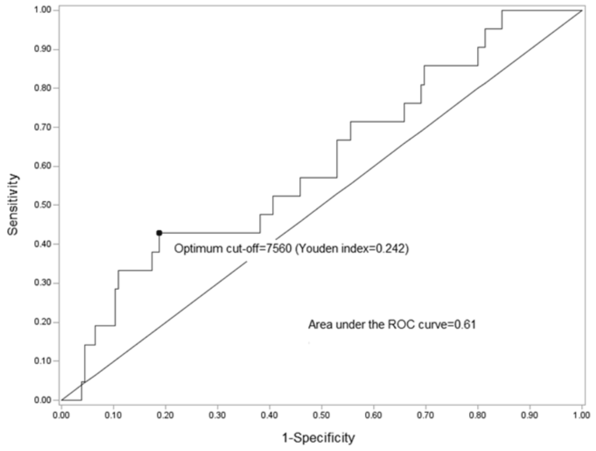

Supplementary Figure S1.
ROC curves associating optimal cut-off values regarding the Youden index for each of Ascitic fluid parameters. Panel A. Protein ascitic fluid count ; Panel B. Glucose ascitic fluid count; Panel C. Lactate Deshydrogenase ascitic fluid count; Panel D. polymorphonuclear leukocytes ascitic fluid count; and Panel E. Leukocyte ascitic fluid count.
